# Towards a Personalized Treatment of Patients with Chronic Myeloid Leukemia

**DOI:** 10.1007/s11899-019-00546-4

**Published:** 2019-11-23

**Authors:** Florence Rabian, Etienne Lengline, Delphine Rea

**Affiliations:** 1 Service Hématologie Adolescents et Jeunes Adultes, Hôpital Saint-Louis, Avenue Claude Vellefaux, 75010 Paris, France; 2 Service d’Hématologie Adultes, Hôpital Saint-Louis, APHP, Paris, France; 3 INSERM UMR 1160, Hôpital Saint-Louis, APHP, Paris, France; 4 France Intergroupe des Leucémies Myéloïdes chroniques (FI-LMC), Institut Bergonié, Bordeaux, France

**Keywords:** Chronic myeloid leukemia, Tyrosine kinase inhibitors, Personalized medicine

## Abstract

**Purpose of Review:**

Treatment goals and ambitions have even been upwardly revised since demonstration was made that under certain conditions, treatment-free remission was possible. Herein, we will discuss on how to try tailoring treatment choices to the unique characteristics of each patient.

**Recent Findings:**

Since the first-generation ATP-competitive TKI imatinib was made available in the clinic in 2001, second-generation drugs such as dasatinib, nilotinib and bosutinib and the third-generation TKI ponatinib have broadened the therapeutic armamentarium, providing effective salvage against intolerance and different types of resistance, or as frontline options.

**Summary:**

Management and outcomes of patients with chronic myeloid leukemia have been revolutionized by the discovery, development, and approval of BCR-ABL tyrosine kinase inhibitors (TKIs). Most patients can now expect a near-to normal life expectancy and acceptable quality of life on life-long treatment, providing awareness and avoidance of harmful adverse events, which depend on each TKI safety profile and patient personal background.

## Introduction

Chronic myeloid leukemia (CML) is a myeloid malignancy characterized by an acquired cytogenetic abnormality in hematopoietic stem cells named the Philadelphia chromosome (Ph1). Ph1 is caused by reciprocal translocation of chromosomes 9 and 22, the t(9;22)(q34;q11). This translocation fuses the breakpoint cluster region (*BCR*) gene on chromosome 22 to the Abelson murine leukemia viral oncogene homolog (*ABL*) gene on chromosome 9. The BCR-ABL oncoprotein, the driver of CML, is a constitutively deregulated intracellular tyrosine kinase that activates a large number of downstream signaling pathways, thereby promoting uncontrolled expansion of genetically unstable myeloid cells [1].

Before the era of therapies targeting the BCR-ABL kinase, most patients died within a few years from the consequences of blast crisis (BC), the terminal phase of the disease. Understanding CML biology as well as BCR-ABL structure and function led to the development and approval in the late 1990s early 2000s of the orally bioavailable first-generation ATP-competitive tyrosine kinase inhibitor (TKI) imatinib. Imatinib shuts down BCR-ABL autophosphorylation and phosphorylation of downstream proteins, leading to the arrest of the pathological signal transduction cascade and leukemic cell death [2, 3]. In the phase 3 “International Randomized Study of Interferon and STI571” (IRIS) trial, rapid reduction in the *BCR-ABL*+ cells pool by imatinib at 400 mg QD in the majority of newly diagnosed chronic-phase (CP)-CML patients translated into a dramatic drop of progression to advanced-phase CML, an outstanding medical breakthrough [4]. The estimated rate of freedom from progression to accelerated phase (AP) or BC was 92.1% at 10 years [5]. In parallel, responses to TKI therapy were recognized as key prognostic markers for long-term outcomes [6••].

Unfortunately, it was rapidly realized that some patients were intolerant to imatinib while others developed resistance and remained at high risk of progression in the absence of salvage option. Resistance is often due to genetic changes such as acquired point mutations in *BCR-ABL* that alter the conformation of the kinase, thereby preventing proper drug binding to the catalytic groove and restoring BCR-ABL activity [7]. For these reasons, second-generation ATP-competitive TKIs with a greater native target inhibitory potency than imatinib, effective control of many kinase domain mutations and different off-target profile were designed. Dasatinib and nilotinib were licensed for patients with resistance or intolerance to imatinib in 2006 and 2007, respectively [8, 9]. Bosutinib, another second-generation drug was approved in 2012 for patients previously treated with at least one TKI and for whom imatinib, nilotinib, and dasatinib are not considered appropriate options [10]. Yet the so-called gatekeeper T315I mutation that completely blocks the access of imatinib and all second-generation drugs to the BCR-ABL ATP-binding site remained a matter of concern. This treatment gap was filled by the third-generation ATP-competitive TKI ponatinib, which displays activity against native and all single mutant forms of *BCR-ABL* including T315I [11]. In 2012, ponatinib received authorization for use in patients with all phases CML and resistance or intolerance to dasatinib or nilotinib and for whom subsequent treatment with imatinib is not clinically appropriate or who have the gatekeeper *BCR-ABL* T315I mutation [12]. Meanwhile, treatment options for newly diagnosed CP-CML expanded after second-generation TKIs were compared with imatinib. Dasatinib and nilotinib were approved in upfront treatment of CP-CML in 2010 and bosutinib in 2017 [13, 14, 15]. Attempts to register ponatinib frontline failed due to unacceptable drug-associated cardiovascular toxicity in this setting [16].

Treatment choice for CML patients used to be straightforward when reduced to imatinib. Since the arsenal of drugs includes second- and third-generation TKIs, physicians face the great challenge of making decision for which drug to start with, when to switch, and which TKI is best on an individual patient basis. Moreover, long-term progression-free survival (PFS) is no longer the sole treatment goal as treatment-free remission (TFR) marks the start of a new era for CML management. In this article, we will address the question of how to personalize CP-CML treatment.

## Tailoring Front-Line Therapy

### Efficacy of TKIs in Newly Diagnosed CP-CML

Second-generation TKIs have been compared with imatinib in the first-line setting, but head-to-head clinical trials allowing direct confrontation between them are lacking. Overall, these drugs allow fewer progression to advanced-phase CML than imatinib and produce higher rates of optimal molecular responses including deep molecular responses (DMR), the latter being a prerequisite for treatment cessation [17] (Table 1).

**Table 1:** Efficacy and frequent AE of first line first and second generation TKIs

Trial	EMR	MMR 1 year	MMR 5 years	DMR 5 years	OS	PFS	Transformation AP/BP	Frequent non-hematological AE^e^
Imatinib								
IRIS	–	27.7^a^	50.3^a^	23^a^	83.3^c^	92^c^	6.9^c^	Fatigue, nausea, diarrhea, muscle cramps, musculoskeletal pain, fluid retention, increase of serum creatinine
DASISION (400 mg QD)	64	28^b^	64^b^	33	90	86	7.7	
ENESTnd (400 mg QD)	69.2	27^b^	60^b^	31	91.7	91	4	
BFORE (400 mg QD)	57.3	36.9^b^	–	–	97.9^d^	6.4^d^	2.5^d^	
Dasatinib								
DASISION (100 QD)	84	46^b^	76^b^	42	91	85	4.6	Pleural effusion, pre-capillary pulmonary arterial hypertension, altered megakaryopoiesis and platelet dysfunction
Nilotinib								
ENESTnd (300 mg BID)	90.7	55^b^	7^b^	54	93.7	92.2	0.7	Metabolic and cardiovascular disorders, ischemic heart disease, cerebrovascular events, peripheral artery disease
Bosutinib								
BFORE (400 mg QD)	75.2	47.2^b^	–	–	99.6^d^	3.7^d^	1.6^d^	diarrhea, vomiting and hepatic transaminase elevation

EMR (early molecular response): BCR-ABL IS% ≤ 10% at 3 months; MMR (major molecular response) : BCR-ABL IS% ≤ 10%; DMR (deep molecular response): response > 4 log reduction

*OS* overall survival, *PFS* progression free survival, *AP/BP* accelerated phase/blast phase, *AE* adverse event

^a^ITT population

^b^Cumulative

^c^At 10 years

^d^At 12 months

^e^Non-exhaustive list

In the “Dasatinib *versus* Imatinib Study in Treatment-Naïve CML Patients” (DASISION) phase 3 randomized trial, early molecular responses (EMR: *BCR-ABL* IS % ≤ 10%) were obtained by 84% of 100 mg QD dasatinib-treated patients and 64% of 400 mg QD imatinib-treated patients [18••]. Cumulative incidences of major molecular responses (MMR: *BCR-ABL* IS % ≤ 0.1%) by 1 year were 46% in the dasatinib arm and 28% in the imatinib arm (*p* = 0.0001). By 5 years, cumulative MMR rates increased up to 76% and 64%, respectively (*p* = 0.0022) and MMR was more rapidly achieved with dasatinib. Although 5-year OS and PFS did not differ between treatment arms, transformation events to AP or BC were lower in the dasatinib arm (4.6%) than in the imatinib arm (7.3%). Moreover, DMR such as molecular response 4.5 (MR4.5: BCR-ABL IS% ≤ 0.0032% or undetectable *BCR-ABL* transcripts with at least 32,000 copies of *ABL* as control) were more frequently attained with dasatinib (42% by 5 years) than with imatinib (33% by 5 years) (*p* = 0.0251).

In the “Evaluating Nilotinib Efficacy and Safety in Clinical Trials-Newly Diagnosed Patients” (ENESTnd) phase 3 randomized study, EMR were achieved by 90.7% of nilotinib 300 mg BID-treated patients and 66.7% of 400 mg QD imatinib-treated [19]. Cumulative incidences of MMR by 1 year were 55% in the nilotinib 300 mg BID arm and 27% in the imatinib arm (*p* < 0.0001), and time to MMR was shorter in the former. By 5 years, cumulative MMR rates were 77% and 60%, respectively (*p* < 0.0001). MR4.5 rates by 5 years were 54% in the nilotinib 300 mg BID arm and 31% in the imatinib arm (*p* < 0.0001). Overall 5-year OS and PFS rates did not differ between nilotinib 300 mg BID and imatinib, but freedom from progression to AP/BC was significantly reduced in nilotinib 300 mg BID-treated patients (99.3%) as compared to imatinib (95.2%) (*p* = 0.0059).

In the “Bosutinib Trial in First-Line Chronic Myelogenous Leukemia Treatment” (BFORE) phase 3 randomized study, a greater proportion of patients treated with 400 mg QD bosutinib reached 3-month EMR as compared to patients receiving 400 mg QD imatinib (75.2% *versus* 57.3%) and MMR rate at 1 year with bosutinib was significantly superior to that obtained with imatinib arm (47.2% *versus* 36.9%) (*p* = 0.02) [15••]. The follow-up is still too short to properly compare PFS and DMR rates between the two treatment arms.

### CML-Related Factors Affecting First-Line TKI Efficacy

The EuropeanLeukemiaNet (ELN) recommends the use of any of the TKIs approved in newly diagnosed CP-CML, considering that there are no sufficiently robust criteria for making the choice [19]. On the other hand, the National Comprehensive Cancer Network (NCCN) clinical practice guidelines in CML suggest that patients with an intermediate or high Sokal or Hasford score at diagnosis may best benefit from second-generation TKIs upfront [20]. In the ENESTnd trial, the 5-year AP/BC rate was substantially reduced in intermediate and high Sokal score patients assigned to nilotinib 300 mg BID (2% and 9%, respectively) as compared to those treated with imatinib (9.9% and 14.1%, respectively) while patients with a low Sokal score had a very low 5-year risk of AP/BC with both TKIs (1% with nilotinib 300 mg BID *versus* 0% with imatinib) [21••]. However, it would be inappropriate to conclude that patients with a low Sokal score do not benefit from second-generation TKIs upfront. Indeed, achievement of DMR is clinically important for patients willing to stop therapy and second-generation TKIs offer higher DMR rates regardless of baseline risk. In ENESTnd, the 5-year cumulative incidence of MR4.5 was 53.4% with nilotinib 300 mg BID *versus* 36.5% with imatinib in patients with a low Sokal score, 60.4% with nilotinib 300 mg BID *versus* 32.7% with imatinib in patients with an intermediate Sokal score, and 44.6% with nilotinib 300 mg BID *versus* 23.1% with imatinib in patients with a high Sokal score [21••].

Apart from baseline risk scores, it is acknowledged that the presence additional chromosomal aberrations (ACA) in Ph1-positive metaphases which occur in about 5% of patients at diagnosis in the CP setting provide higher likelihood of progression when imatinib is chosen upfront, especially trisomy 8, trisomy 19, isochromosome 17, Philadelphia chromosome duplication, monosomy 7, or 3q26.2 rearrangements [22]. Whether the poor prognostic significance of these baseline ACA persists in patients receiving first-line or second-generation TKIs needs to be explored. It is thus difficult to firmly guide treatment choice based on this sole parameter.

### Safety of First-Line TKIs and Patient-Related Factors

All TKIs display distinct adverse event profiles, possibly reflecting differences in their off-target activities (Table 1) [23]. Imatinib at the standard 400 mg QD dose rarely leads to severe injuries, but mild to moderate non-hematological toxicities such as fatigue, nausea, diarrhea, muscle cramps, musculoskeletal pain, and fluid retention are often chronic and may impair quality of life and adherence [24]. Although long-term use of imatinib is considered as safe, concerns have been raised about a potential renal damage as a rise in serum creatinine overtime has been reported [25]. Whether this is simply due to the inhibition of tubular creatinine secretion by imatinib, to a true drug-associated glomerular damage or to confounding causes of chronic kidney disease such as aging, diabetes, or hypertension has not been fully solved [26].

Main issues associated with nilotinib use consist in an excess risk of metabolic and cardiovascular disorders [23, 27]. In the ENESTnd trial, the incidence of ischemic heart disease, cerebrovascular events, and peripheral artery disease increased overtime in the nilotinib 300 mg BID arm and reached 7.5% by 5 years *versus* 2.1% in the imatinib arm [21••]. Such events are clearly influenced by individual background, patients already at high or very high cardiovascular disease (CVD) risk being the most vulnerable [28], [21••]. Nilotinib also impairs glucose and cholesterol homeostasis through mechanisms that are not completely understood [29] [30]. These parameters need to be tightly monitored and controlled throughout treatment as diabetes and dyslipidemia are well-known risk factors for CVD.

Iatrogenic lung damage is among the most common non-hematological toxicities linked to dasatinib administration. In the DASISION trial, pleural effusions were experienced by 28% of patients assigned to the dasatinib 100 mg QD arm by 5 years and occurred in about 6–9% of patients at risk annually, with continuous risk overtime [18••]. These may develop through an immune mechanism as suggested by their lymphocytic and exudative nature. Pleural effusions typically resolve upon dasatinib interruption but the risk of recurrence is noticeable when treatment is reintroduced. Older age is the main risk factor for developing pleural effusion [31]. In DASISION, pleural effusion occurred in 60% of patients aged ≥ 65 years and 25% in patients below 65 [18••]. Physicians should also be aware of pre-capillary pulmonary arterial hypertension (PAH), a very rare but potentially fatal complication of dasatinib therapy [32]. No risk factors for PAH have been identified; PAH may be totally or partially reversible after dasatinib withdrawal and permanent discontinuation of dasatinib is mandatory. Finally, altered megakaryopoiesis and platelet dysfunction have been described with dasatinib; thus, caution is needed in case of thrombocytopenia or antiplatelet therapy as major bleeding may occur [33][ [34].

Gastrointestinal and hepatic disorders represent the hallmark of bosutinib toxicity. Initial attempts to register bosutinib as frontline treatment failed when the drug was assessed at 500 mg QD. With 400 mg QD, the actually approved dosage of first-line bosutinib, 70.1% of patients in the BFORE trial reported all grades diarrhea (grade ≥ 3 7.8%) *versus* 33.6% (grade ≥ 3 0.8%) of patients treated in the imatinib control arm [15••]. All grades elevation of liver enzymes occurred in 39.9% (grade ≥ 3 24.3%) of patients receiving bosutinib and in 13.6% (grade ≥ 3 4.2%) of patients receiving imatinib.

### Making First-Line Treatment Choices

The question of which generation of TKI to start with is important, as chance of reaching optimal end points in a timely fashion is undeniably greater with second-generation TKIs than with imatinib. However, second-generation TKIs at their currently approved doses may expose some patients to toxic effects on vital organs, especially elderly populations and those with comorbidities. We must also recognize that second-generation TKIs have not been able to demonstrate an OS advantage over first-line imatinib, but this may be due to the availability of salvage options. Nevertheless, goals of CML treatment have markedly evolved over the past few years. Durable TFR in patients with long-lasting DMR is widely recognized as an achievable reality, and second-generation TKIs offer greater opportunity of TKI discontinuation eligibility than imatinib [35]. TFR may not only be appealing to the youngest but also to those for which long-term TKI exposure is not desirable for safety, financial, or personal reasons. It is important to realize that CML incidence remains stable between 1 and 2 cases per 100, 000 inhabitants per year in the USA and Europe, but prevalence is steadily rising [36]. Thus, TFR also represents a great opportunity to mitigate the rise in healthcare expenditures on CML drugs.

**Fig. 1:**
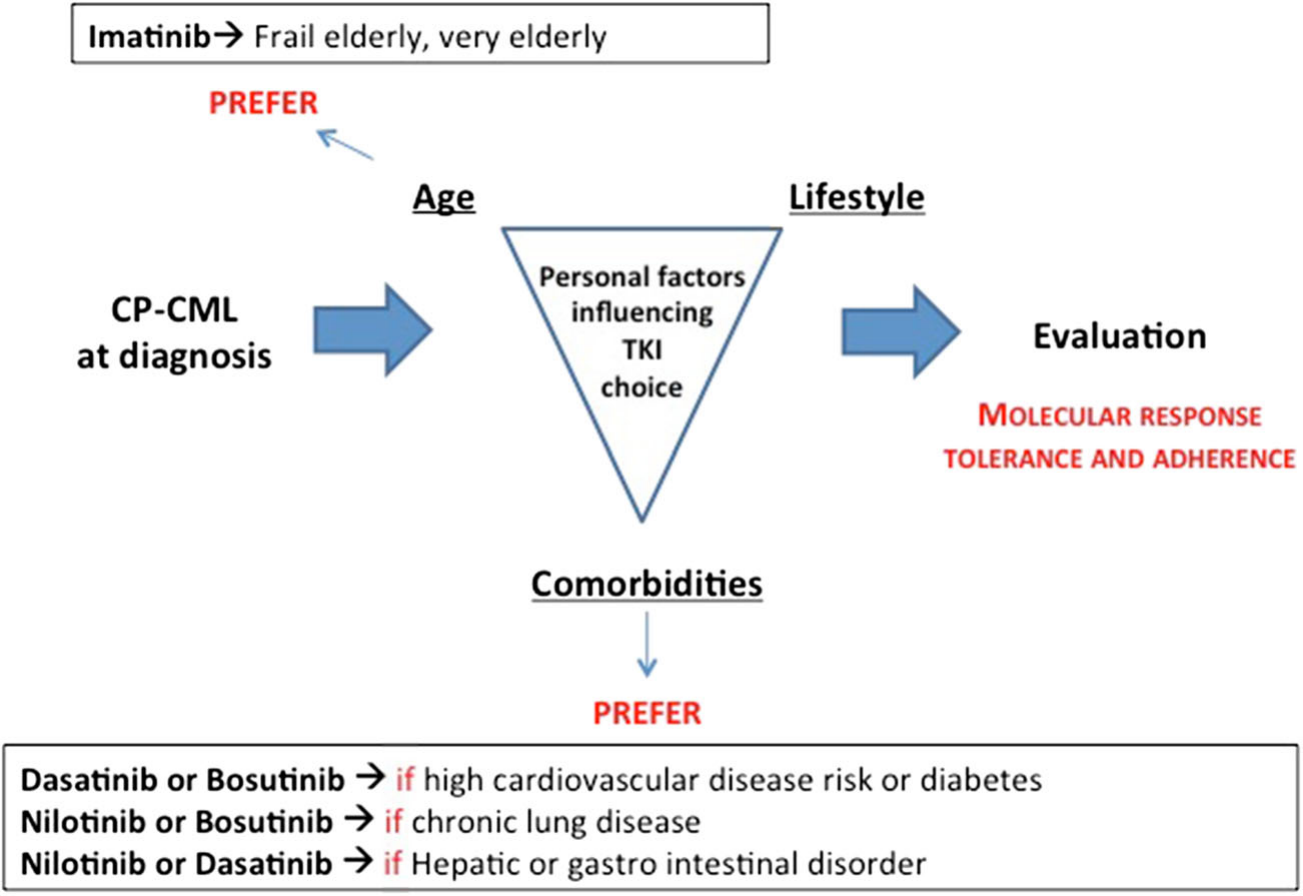
Patient-related factors to be taken into account when making first-line treatment choice

Seeking for the best possible care and outcome including TFR, our position is to opt for second-generation TKIs upfront in all newly diagnosed CP-CML patients whenever affordable and available, regardless of baseline prognostic factors. Notable exceptions reside in the presence of what we consider prohibitive personal factors (Fig. 1). We still prefer imatinib in geriatric patients who are more prone to comorbidities and iatrogeny. We usually avoid first-line nilotinib in patients at very high cardiovascular risk and in diabetic patients, regardless of age. When nilotinib is chosen, we implement longitudinal cardiovascular disease risk prevention strategies proven to be effective in the general population [37]. We refrain from choosing first-line dasatinib in elderly patients or those with underlying chronic lung disease. Finally, it may be wise to keep away from first-line bosutinib in patients with active hepatic disease or chronic gastrointestinal disorders such as uncontrolled inflammatory bowel disease, although there are no data to strongly support this statement as such profiles were excluded from clinical trials. Finally, ease of administration is quite poor with nilotinib due to significant high-fat food effect on drug bioavailability and a BID schedule; thus, patient preference and lifestyle may impact drug choice. Regardless of which TKI is chosen frontline, treatment failure gathering intolerance or resistance should be recognized early as a prompt intervention increases the chance of achieving best possible global outcome.

## Tailoring Strategy After Front-Line Therapy

### Response-Driven Treatment Changes

During TKI treatment, the degree to which the bulk of leukemia is reduced is a key prognostic marker of PFS. Accurate quantification of residual disease is necessary to guide clinical decisions. Ideally, every patient should have access to regular molecular monitoring according to a well-defined surveillance program and reliable internationally standardized quantitative PCR methods [38]. Assessment of molecular responses is also indispensable to identify candidates for TKI discontinuation. Optimal responses enable a near-to normal life expectancy and do not require any therapeutic modification in the absence of safety issue, unless patients qualify for stopping treatment [20] [39] [40].

Identification of resistance necessitates a change in treatment strategy, primarily guided by results from *BCR-ABL* mutational analyses. In the absence of a *BCR-ABL* mutation, any second-generation TKI may be chosen in patients resistant to first-line imatinib although it is somewhat unfortunate that no direct comparison studies were performed [20] [19]. For those resisting a first-line second-generation TKI, ponatinib may be a more efficient option than another second-generation TKI but this has regrettably not been investigated in a randomized study [41]. In case of resistance to a second-line second-generation TKI, several lines of evidence point to greater results with ponatinib than with an alternate second-generation TKI [42]. Ponatinib at the registered dose of 45 mg QD exposes patients to a high burden of arterial occlusion events especially when atherosclerotic CVD or strong risk factors like diabetes or hypertension are already present; thus, CVD prevention is essential [12]. Ponatinib-associated newly occurring or worsening hypertension is another matter of concern, and it must be detected early as it can be controlled with dose adjustment and anti-hypertensive drugs [12] [16].

In the presence of a *BCR-ABL* mutation, selection of the most effective alternate TKI is possible, depending on localization within the different structural and functional domains of the kinase, *in vitro* sensitivity, and clinical efficacy data. Over 100 different mutations have been discovered in imatinib-resistant patients while the spectrum of mutations resistant to second-generation TKIs is much narrower. With the exception of T315I, mutations that confer resistance to nilotinib or dasatinib hardly overlap. Nilotinib is ineffective against *BCR-ABL* mutations Y253H, E255K/V, and F359V/C/I, both *in vitro* and in patients [43]. Of note, the initial recommended dose of nilotinib second line or beyond is 400 mg BID, substantially higher than what given first line. Awareness of the dose-dependency of cardiovascular toxic effects of nilotinib and vigilance is essential in order to avoid irreversible complications [21••]. The *BCR-ABL* mutations V299L, T315A, and F317L/V/I/C confer a high degree of resistance to dasatinib *in vitro* and dasatinib fails to rescue harboring those [44]. The *BCR-ABL* mutations E299V, G250E, E255K are associated with high or very high resistance to bosutinib both *in vitro* and in patients [45]. The T315I mutation is sensitive to ponatinib only and the prognosis of patients carrying this mutation impressively improved since approval of ponatinib. Five-year report of phase 2 “Ponatinib Ph1 ALL and CML Evaluation” (PACE) international study indicated a 70% rate of complete cytogenetic responses, a 58% rate of MMR, and a 38% rate of MR4.5 in the T315I+ cohort and responses were sustained, thus dispelling the specter of allogeneic stem cell transplantation (ASCT) [46] [47]. Although exceptionally performed, ASCT remains a key option in case of multi-resistance or progression to AP/BC.

Warning corresponds to a situation where TKIs decrease *BCR-ABL* transcripts below the 1% IS threshold during the first year of therapy, but MMR is not attained. Patients in the warning zone need to be carefully monitored as secondary resistance may finally emerge [6]. Alternatively, the molecular response may remain stable overtime or even spontaneously improve with longer duration of treatment; thus, a change in TKI is not absolutely required [20] [19]. However, in the absence of a switch to a more potent TKI, the likelihood of a DMR for patients in the warning zone is quite poor [48] [49], precluding any TKI discontinuation attempt. A switch from first-line imatinib to a second-generation TKI appears as an interesting option for those aiming at TFR. The randomized phase 3 “ENEST–Complete Molecular Remission” study investigated the probability to gain a DMR upon transition to nilotinib. For patients in the warning zone on long-term imatinib, the probability to achieve a MR4.5 was 33.3% by 4 years in the nilotinib 400 mg BID arm *versus* 3.6% for in the imatinib control arm [50•]. Earlier switch based on warning at specific time points during the first year of imatinib therapy may provide even better results as suggested by results from the “Therapeutic Intensification in De Novo Leukaemia (TIDEL)-II” study [51]. What to do for patients in the warning zone on first-line second-generation TKI is not straightforward. In our opinion, ponatinib is not an option to address such situation as risks of switching may outweigh benefits.

### Toxicity-Driven Treatment Changes

Severe, recurrent, or chronic toxicity forces discontinuation of the relevant TKI unless manageable by supportive care or dose reduction. General rule for next TKI choice includes avoidance of cross-intolerance while efficacy should remain in focus. Cross-intolerance relates to the recurrence during treatment with a new TKI of the same adverse event that led to intolerance to the prior TKI. Pooled data from several clinical trials showed that among patients with severe non-hematological intolerance to imatinib, cross-intolerance with dasatinib was observed in 4% of the cases, mainly including skin rash, myalgia, and arthralgia [52]. Analysis of data from the pivotal phase 2 registration trial of nilotinib 400 mg BID showed that 7% of patients with severe or moderate but chronic non-hematological intolerance to imatinib developed side effects of the same nature on nilotinib, mainly including diarrhea [53]. Of note, there are some data to support the use of 300 mg BID of nilotinib instead of 400 mg BID in patients responding well but intolerant to first-line imatinib or dasatinib [54] [55]. Rate of cross non-hematological intolerance between imatinib and bosutinib was considered as low during development of bosutinib at 500 mg QD beyond the first-line setting, with the notable exception of gastrointestinal disorders such as nausea and diarrhea [45]. However, pleural effusion on bosutinib may occur at a very high frequency in patients with such a history on prior TKI, especially dasatinib [56]. Finally, it may not be wise to choose ponatinib in patients experiencing arterial occlusion on nilotinib for obvious safety reasons, except in patients deemed at high risk of disease progression in the absence of a suitable alternative option.

## Conclusion and Perspectives

By enabling each patient to benefit from CML risk assessment, profiling of extent, and severity of co-morbidities and molecular monitoring during treatment, physicians have the possibility to select TKIs on an individual basis to ensure minimal harmful side effects and maximum successful outcome. Despite this impressive accomplishment, a number of challenges remain, such as more accurate CML prognostication, safer dosing options of TKIs, multi-resistance to therapy, expanded eligibility to and better outcome of TKI discontinuation. As somatic mutations outside the *BCR-ABL* gene may be present in CML, investigation of a patient’s genetic and epigenetic landscape with the use of modern sequencing technologies in order to improve existing CML risk prediction models not only at the time of diagnosis but also in case of resistance or progression and help choosing or developing treatments that are most likely to be successful is worth exploring [57]. Advances in the past 20 years with TKIs being the cornerstone of treatment have helped transform CML from a fatal malignancy to a chronic condition, and safety should be placed at the forefront of management together with efficacy. Following drug development and health agencies approval, the recommended dosage of TKIs consists in a standard one-size-fits-all dose, with dose adjustments solely envisaged on tolerance issue. We believe that it is time to revisit this conservative strategy in the era of personalized treatment. Maintenance with low doses of TKIs after achievement of an optimal response appeared safe and beneficial in the phase 2 “De-Escalation and Stopping Treatment with Imatinib, Nilotinib, or sprYcel” (DESTINY) [58]. Our team evaluates conversion to low-dose nilotinib after MMR achievement with a standard dose, and preliminary results point in the same direction [59]. Doses lower than the currently approved ones as frontline therapy in newly diagnosed CML patients are also investigated as new potential standard-of-care option [60]. Last but not least, allosteric BCR-ABL inhibitors, a new class of highly selective TKIs, are being evaluated in clinical trials. These inhibitors bind the myristoyl-binding pocket of BCR-ABL distantly located from the ATP-binding site and restore auto-inhibition mechanism of the kinase [61]. Whether these alone, combined to ATP-competitive TKIs or to CML stem cell targeting agents will have the potential to safely rescue, bring TFR, or even cure to more patients including those with a history of resistance is an open question.
